# Profil épidémiologique des hémoglobinopathies: étude transversale descriptive autour du cas index

**DOI:** 10.11604/pamj.2017.27.150.11477

**Published:** 2017-06-29

**Authors:** Fatima Dahmani, Souad Benkirane, Jaafar Kouzih, Aziz Woumki, Hassan Mamad, Azlarab Masrar

**Affiliations:** 1 Équipe de Recherche en Hématologie, Laboratoire d'Hématologie, Faculté de Médecine et de Pharmacie, Université Mohammed V, Rabat, Maroc; 2 Laboratoire Central d'Hématologie, Centre Hospitalier Ibn Sina, Rabat, Maroc

**Keywords:** Epidémiologie, thalassémies, drépanocytose, anémie, Kenitra, Maroc, Epidemiology, thalassemias, sickle cell disease, anaemia, Kenitra, Morocco

## Abstract

Les hémoglobinopathies sont des affections constitutionnelles conséquentes à des anomalies des hémoglobines. Elles sont souvent graves dans leurs formes majeures, leur prise en charge est lourde avec un grand impact psycho-social sur les patients et leur famille. Classées parmi les maladies rares, elles sont encore insuffisamment connues des professionnels de santé. Cette méconnaissance est à l'origine d'une errance diagnostique, d'un retard dans leur prise en charge et par conséquent une morbidité et une mortalité élevée chez ces patients. L'Organisation Mondiale de la Santé (OMS) a publié en 2008 des données concernant l'épidémiologie des hémoglobinopathies: plus de 330000 cas naissent chaque année avec une hémoglobinopathie (83% des cas de drépanocytose, 17% des cas de thalassémie). Les troubles de l'hémoglobine sont responsables d'environ 3,4% des décès chez les moins de 5 ans. A l'échelle mondiale, 7% environ des femmes enceintes seraient porteuses d'une forme de la thalassémie et 1% des couples sont à risque. Toutefois, elles sont relativement fréquentes dans certaines régions du globe où les mariages consanguins sont communs. Afin de décrire les caractéristiques épidémiologiques des familles à risque d'hémoglobinopathies (étude autour du cas) dont les cas index sont suivis au service de pédiatrie à l'Hôpital Provincial El Idrisi de Kenitra au Maroc, une étude transversale descriptive a été réalisée durant deux enquêtes la première en mai 2015 et la deuxième en juin de la même année lors des journées de vaccination des cas index contre le pneumocoque. Après avoir recueilli les données épidémiologiques de nos patients, nous avons réalisé une étude biologique comportant: l'hémogramme avec étude morphologique des globules rouges en coloration MGG et numération automatique des réticulocytes; les électrophorèses de l'hémoglobine à pH alcalin (8.8) et secondairement à pH acide (5.4) sur gel d'agarose avec intégration densitométrique. 275 patients ont présenté des profils compatibles à une hémoglobinopathie. La majorité de ces malades étaient issus de mariages consanguins (83.1%) et originaires de régions situées dans le nord du Maroc. L'enquête familiale a permis de retrouver les familles à risque, chez lesquelles on note une fréquence élevée de drépanocytose. Nos résultats confirment l'existence de différents types d'hémoglobinopathies dans la population marocaine.

## Abstract (en)

Hemoglobinopathies are congenital disorders resultimg from hemoglobin abnormalities. Major forms are often severe, their management is difficult and associated with a great psychosocial impact on patients and their families. They are classified as rare diseases and are still insufficiently known by health professionals. This lack of knowledge is at the origin of diagnostic errors, delay in their management and therefore high morbidity and mortality rate for these patients. In 2008, the World Health Organization (WHO) has published data on hemoglobinopathies epidemiology: more than 330.000 cases of hemoglobinopathy occur each year (83% of cases of sickle cell anemia, 17 % of cases of thalassemia). Hemoglobin disorders are responsible for approximately 3.4% of deaths among people under the age of 5. At the global level, approximately 7% of pregnant women would be carriers of a form of thalassemia and 1% of couples are at risk. However, they are relatively frequent in some regions of the globe where consanguineous marriages are common. We conducted a descriptive cross-sectional study based on two surveys, the first in May 2015 and the second in June of the same year. It was performed in the immunization days to deliver pneumococcal vaccine to the index cases and it was aimed to describe the epidemiological features of families at risk of hemoglobinopathies (index case study), whose index cases were treated in the Department of Pediatrics at the Provincial Hospital El Idrisi, Kenitra, Morocco. After having collected the epidemiological data from patients, laboratory tests were performed including: blood count with red blood cells morphological assessment using the MGG assay and automatic numbering of reticulocytes; hemoglobin electrophoresis at alkaline pH (8.8) and then at acid pH (5.4) on agarose gel and densitometric integration. 275 patients had laboratory profiles compatible with hemoglobinopathy. The majority of these patients were born to consanguineous marriages (83.1%) and came from the north regions of Morocco. This family survey allowed to identify families at risk with a high frequency of sickle cell anemia. Our results confirm the existence of hemoglobinopathies variants among Moroccan population.

## Introduction

Au Maroc, l'épidémiologie des hémoglobinopathies reste inconnue. L'OMS estime le taux des porteurs au Maroc à 6.5%, ce qui laisserait supposer l'existence de 30.000 cas de formes majeures de thalassémie et drépanocytose au Maroc [1]. Plusieurs études menée par l'hôpital d'enfant du centre hospitalier universitaire (CHIS) ont montré que le Nord-Ouest du Maroc est une zone de prédilection des hémoglobinopathies et que la région de Rabat Salé Kenitra, semble la région la plus touchée plus particulièrement au niveau de la province de Kenitra qui constitue un foyer riche d'hémoglobinopathies [2]. Consciente de cette situation, l'OMS a déclaré dans plusieurs assemblées l'urgence pour les pays touchés de concevoir et de mettre en œuvre des programmes nationaux intégrés de prévention et de prise en charge de la thalassémie, drépanocytose et les autres hémoglobinopathies [3]. Ainsi, nous nous sommes proposés de réaliser une étude dans le but de déterminer profil des hémoglobinopathies chez une population à risque (cas index et sa famille) de la région Rabat Salé Kenitra, afin de contribuer à une meilleure prise en charge des familles touchées par ces désordres et contribuer à l'aide des décideurs à proposer des stratégies appropriées de prévention et de prise en charge de ces pathologies.

## Méthodes

**Figure 1:**
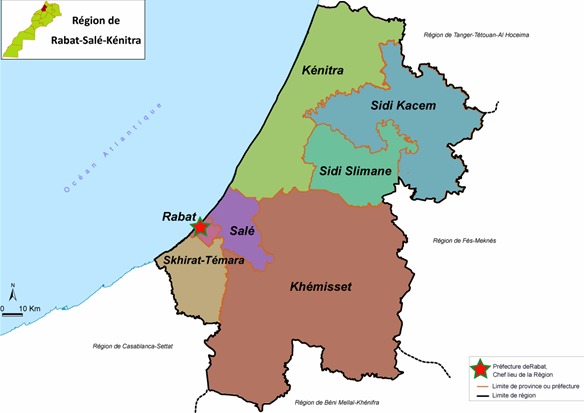
Situation géographique de la région rabat Salé Kenitra

Il s'agit d'une étude transversale descriptive des cas index suivis au service de pédiatrie de l'Hôpital Provincial El Idrissi de Kenitra, et leurs familles. Les familles ont étés informés du but de l'enquête, ceux de 100 familles (soit 3.79/famille) ont étés dénombrés et répertoriés. L'Hôpital est situé dans la région du Gharb Chrarda Beni Hssen du Maroc. Cette région du Gharb située au Nord-Ouest du Maroc à proximité de l'Océan Atlantique entre Rabat et Tanger. Le climat est de type méditerranéen, caractérisé par une alternance d'une saison humide d'Octobre à Avril et une saison sèche et chaude de Mai à Septembre. Sa superficie est de 4200 KM, sa population est estimée à 1859540 habitants, selon le dernier recensement général de la population et de l'habitat (RGPH-2004) (Figure 1).

### Interrogatoire

Pour chaque patient, nous avons recueillies renseignements démographiques, à savoir l'âge, le sexe, l'origine géographique, les antécédents personnels et/ou familiaux et d'éventuels notions de consanguinité.

### Exploration hématologique

Les critères diagnostics appliqués dans cette étude sont un hémogramme anormal, frottis sanguin présentant des anomalies érythrocytaires et un profil électrophorétique correspondant à une hémoglobinopathie.

### Etape pré-analytique

Les prélèvements ont été effectués, pour chacun des patients de notre étude, sur tube anticoagulé (EDTA) pour l'hémogramme et l'étude de l'hémoglobine.

### Etape analytique

L'hémogramme a été réalisé sur automate Sysmex XE 5000. Les électrophorèses de l'hémoglobine à pH alcalin (8.8) et secondairement à pH acide (5.4) sur gel d'agarose sur un équipement Interlab pretty. La quantification des fractions de l'hémoglobine sur un densitomètre epson perfection V700 Photo.

### Saisie, analyse et traitement des données

Les données ont été saisies sur excel et analysées par un logiciel statistique version 10 de windows. Les variables quantitatives ont été exprimées en moyenne et en écart type et les variables qualitatives en nombre et en pourcentage. Pour les tests de comparaison, nous avons utilisé les tests X^2^. Une valeur de p inférieure à 0.05 était considérée comme significative.

### Considérations éthiques

Les travaux liés au manuscrit que je m'apprête à soumettre ont reçus l'approbation des autorités administratives de toutes les institutions impliquées. Le consentement éclairé (verbal ou écrit) pour la participation à l'étude est obtenue avant le recrutement de chaque famille participante. En effet, l'objectif, la méthodologie, les avantages attendus et les contraintes de toutes les procédures prévues ont été décrites brièvement puis une feuille de consentement a été signée par chaque famille participant à l'étude. Les familles qui ont participé à l'étude ont obtenu un bilan hématologique et le profil de leurs hémoglobines. Celles qui sont porteuses d'hémoglobinopathies ont bénéficié de conseils et d'une orientation pour le suivi et la prise en charge. Des dispositions pratiques de préservations des informations ont été mises en œuvre pour assurer la confidentialité des données. Notamment, l'engagement de toutes les personnes participant à la réalisation de l'étude à garder confidentielles les informations recueillies a été assuré. Les résultats de l'étude ont permis de déterminer le profil épidémiologique des hémoglobinopathies ce qui aidera les décideurs à proposer des stratégies appropriées de prévention et de prise en charge de ces pathologies.

## Résultats

Nous avons diagnostiqué au total 275 patients atteints d'hémoglobinopathies. Les paramètres démographiques et les différentes hémoglobinopathies recensées en fonction des résultats d'électrophorèse d'hémoglobine sont présentés dans les Tableau 1 et Tableau 2. Au total, 104 patients (27.4%) avaient une électrophorèse d'hémoglobine normale. Les type d'hémoglobinopathies recensées incluaient la drépanocytose hétérozygote AS (40.6 %); la drépanocytose homozygote SS (23%); les traits ß-thalassémie: (3.2%); l'hétérozygotie composite S/ß-thalassémie (2.9 %); l'hémoglobinose AC (2.4 %), la double hétérozygotie SC et la a-thalassémie mineure (0.3% respectivement).

**Tableau 1:** Etude descriptive de la population

Caractéristique	Valeur
Age (en année)*	14 (8-33)
Sexe	
Masculin	179 (47,2)
Féminin	200 (52,8)
Consanguinité	
Oui	315 (83,1)
Non	64 (16,9)
Hémoglobinopathies	
Normal	104 (27,4)
Drépanocytose hétérozygote A/S	154 (40,6)
Drépanocytose hétérozygote S/S	87 (23)
Trait β-thalassémie	12 (3,2)
Hétérozygotie composite S/ β-thalassémie	11 (2 ,9)
Hémoglobinose hétérozygote AC Double hétérozygotie SC	9 (2,4) 1 (0.3)
α-thalassémie mineur	1 (0,3)

* Exprimé en médiane et intervalle interquartiles

Exprimé en effectif et pourcentage

**Tableau 2:** Les différentes hémoglobinopathies recensées en fonction des résultats d’électrophorèse d’hémoglobine

	Nombre de cas	Hb A	Hb A2	Hb F	Hb S	HbC
Normal	104	97± 2.04	2,3± 1,5	<1		
Trait β-thalassémie	12	93.3±3.42	5.75±1.8	1.06±2.63		
Hémoglobinose A/C	9	60±2.5	3±2.3	0.4±0.8		36.6±2.3
α-thalassémie mineur	1	±	±	±	±	±
Drépanocytose hétérozygote A/S	154	61.7±5.2	2.5±0.6		35.8±1.3	
Hétérozygotie composite S/ β-thalassémie	11	11.9±17.9	4.36±1.9	16.83±23.9	63±18.5	
Hétérozygotie composite S/C	1		2.7±0.8	1.2±0.4	49.4±3.5	47.7±5.3
Drépanocytose homozygote S/S	87		2±0.7	9.1±2.3	88.9±4.9	

### Aspect épidémiologiques

**Age:** L´âge au moment du diagnostic variait de 3 mois à 54 ans, avec un âge médian de 8 ans pour l'enfant et 33 ans pour l'adulte. La répartition des cas par tranche d'âge au moment du diagnostic est représentée dans le Tableau 3.

**Tableau 3:** Répartition des patients par tranche d’âge au moment du diagnostic

Tranche d'âge en années				
	0-5 ans	6-10 ans	11-16 ans	>16 ans
Normal	17	16	12	59
	16.3%	15.4%	11.5%	56.7%
Drépanocytose heterozygote A/S	23	20	16	95
	14.9%	13%	10.4%	61.7%
Drépanocytose homozygote S/S	32	22	19	14
	36.8%	25.3%	21.8%	16.1%
Trait bétathalassémie	3	4	1	4
	25%	33.3%	8.3 %	33.3%
Hétérozygotie composite S/ β-thalassémie	2	4	2	3
	18.2%	36.4%	18.2%	27.3%
Hémoglobinose hétérozygote AC	4	3	0	2
	44.4%	33.3%		22.2%
Hétérozygotie composite S/C	1	0	0	0
	100.0%			
Alpha thalassémiemineur	0	0	1	0
			100.0%	
Total	82	69	51	177
	21.6%	18.2%	13.5%	46.7%

**Sexe**: 179 patients étaient de sexe masculin et 200 de sexe féminin avec sex-ratio de 0.9 (Tableau 1).

**Répartition géographique**: Les cas étudiés sont tous originaires des régions Rabat Salé Kenitra.

**Consanguinité**: De cette même population, 315 patients (83.1%) sont issus d´un mariage consanguin (Tableau 1).

**Aspect biologiques**: Une anémie normocytaire normochrome est observée chez 70% des patients. Les autres patients présentent une anémie microcytaire hypochrome. Le degré d'anémie est très variable, sévère dans les formes majeures de drépanocytose homozygote S/S (88.5%), chez les formes composites S/ß thalassémiques (100%), et modérée chez les hétérozygoties A/C (77.8%), et le trait ß thalassémie (75%). Le degré d'anisocytose est liée au degré d'anémie, très évocatrice chez les drépanocytaires homozygotes S/S (95.4%), les formes composites S/ß thalassémies, les hétérozygoties A/C et le trait ß thalassémie. Une réticulocytose était observée chez les drépanocytaires homozygotes S/S (81.6%), S/ ß thalassémiques (90.9%) (Tableau 4).

**Tableau 4:** Paramètres de l’hémogramme chez la population étudiée

Paramètres	Groupe A/C n=9	Groupe A/S n=154	Groupe S/S n=87	Groupe S/ β Thalassémie n=11	Groupe trait et β thalassémie n=12
Hémoglobine (g/dL)	9.25 ± 3.11	12.13± 2.44	7.59 ± 0.91	8.41± 2.13	10,23 ± 2.04
VGM (μm3)	63.42 ± 9.23	77.32 ± 8.13	80.81±12.37	78.69 ± 11.90	74.10 ± 15.19
TCMH (pg)	21.56 ± 3.48	26.87± 3.20	28.0273 ±4.50	27.77± 3.89	24.02± 5.01
CCMH (%)	33.97 ± 1.42	34.70 ± 1.62	34.6545± 1.04	34.88 ± 1.69	32.42 ± 1.95
Réticulocytes (Giga/L)	45 ,5 ± 11,76	80 ± 86	363 ± 195,5	262 ± 134	117 ± 164
Plaquettes (Giga/L)	297.22 ± 156,58	425.39±148.47	335.90±101,11	319.56±125.77	288± 106,11
Leucocytes (Giga/L)	8.67 ± 2.9585	7.98 ± 3.98	13.55± 5.32	13.34 ± 5.28	9.61 ± 3.95
Neutrophiles (Giga/L)	4.0500 ± 1,69	5.48± 2.44	5.028± 1,86	3.61± 2.34	4,89± 3,64
RDW (%)	18.1222± 2,82	20.18± 3.98	19.34± 4,47	15.17± 3.13	19,75± 6,99

VGM: volume globulaire moyen; CCMH: concentration corpusculaire moyenne en hémoglobine ; TCMH : Teneur corpusculaire moyenne en hémoglobine

**Les aspects morphologiques**: Chez les sujets hétérozygotes A/S et A/C le frottis sanguin ne présente pas de particularités, chez les formes sévères S/S et les hétérozygoties composites S/Béta thalassémie, le frottis sanguin montre une poikylocytose, des cellules cibles, des drépanocytes, dans les cas particuliers de l'hétérozygotie composite S/C on retrouve sur le frottis des cellules cibles et des drépanocytes.

## Discussion

Ces premiers résultats, bien qu'intéressant 379 cas d'une population à risque, présentent une importante valeur d'information sur la prévalence des hémoglobinopathies au Maroc et que la drépanocytose constitue l'anomalie majeure de l'hémoglobine dans notre pays et donc un problème de santé publique. Pour chacun de nos 379 patients, nous avons retenu l'âge, le sexe ainsi que le profil électrophorétique. La moyenne dâge de nos patients était de 14 ans (limites 3 mois et 54 ans)avec un âge médian de 8 ans pour l'enfant et 33 ans pour l'adulte. En effet la stratification du groupe étudié se rapproche d'une pyramide d'âges standards de la population marocaine, avec un pourcentage élevé d'enfants. La jeunesse de notre population a été remarquable car 53.3 % avaient moins de 16 ans. L'âge relativement jeune pourrait s'expliquer par la précocité des manifestations cliniques de certaines maladies de l'hémoglobine [4]. Un résultat similaire a été observé par Haidara [5] qui trouvait que 50% de sa population avaient moins de 30 ans et souffraient de drépanocytose alors que coulibaly [6] trouvait que 42.2% de sa population avaient moins de 31 ans et souffraient par contre de l'hémoglobinose C. Un déséquilibre du sex-ratio en faveur du sexe féminin qui était relativement plus intéressé (179 sujets de sexe masculin pour 200 sujets de sexe féminin). Ce sex-ratio qui de 0.9 est partiellement expliqué par le biais de l'échantillonnage et le mode de recrutement. Cette disparité reflète l'absence des hommes souvent moins concernés par une enquête de santé, que les mères de famille. Par ailleurs, on ne s'attendait pas à une disproportion de répartition entre les deux sexes compte tenu du mode de transmission génétique des anomalies de l'hémoglobine: mode autosomique récessif [7, 8]. Les cas étudiés sont tous originaires de la région Rabat Salé Kenitra qui est la région la plus touchée au Maroc, anciennement impaludée, ce dernier exerce une pression sélective par procuration d'un avantage de survie aux porteurs des hémoglobinopathies [9, 10], ceci peut expliquer le succès de la mutation drépanocytaire. Cette région se singularise aussi par un très haut degré de consanguinité. Le Maroc reste un pays très enraciné dans ses traditions et coutumes et l'endogamie en est la preuve. Les signes cliniques les plus fréquemment observés isolés ou associés étaient: douleurs, anémie, ictère et splénomégalie. Toutes ces altérations peuvent être minimisées par un diagnostic précoce.

Sur le plan biologique, presque la moitié des formes se traduisent dans notre étude par une anémie normocytaire 59.1%, la microcytose observée chez certains patients drépanocytaires 40.1%, oriente vers l'association avec une carence martiale, une a thalassémie, une hétérozygotie composite ou un autre mutant de l'hémoglobine. En effet on n'avait pas fait de bilan martial pour expliquer la microcytose trouvée. Ce phénomène, d'anémies microcytaire hypochromes est très souvent rapporté dans la littérature pour les hémoglobinopathies [11, 12]. L'anisocytose est très marquée dans ces formes. L'anémie d'intensité variable. Elle est très régénérative dans La forme S/ß thalassémie et la drépanocytose homozygote. Ceci est en accord avec les études de Nacoulma, Tshiloto et Omoti [13, 14]. Ces formes même en dehors des crises ont continuellement une hémolyse des globules rouges [15]. Quelques patients drépanocytaires SS de notre étude étaient sous acide folique, mais il était difficile de s'assurer de son prise adéquate étant donné que le médicament était pris chez eux. Cependant, Bazuaye observe une différence du taux d´hémoglobine chez le drépanocytaire sous acide folique déjà après 14 jours de traitement [16]. Une hyperleucocytose constante est retrouvée dans notre étude, les hémoglobinopathies sont des maladies inflammatoires dont l'un des marqueurs est la leucocytose [17]. En effet les infections répétées exposent les patients malades à de nombreux risques. Des réactions immunologiques variées peuvent survenir, dans n'importe quel contexte de transfusions [18, 19]. Un taux élevé de leucocytes est associé à un risque élevé de décès précoce [20]. Le nombre moyen de plaquettes dans notre étude est celui rapporté dans la littérature [15]. Les études de Jaffe à Ontario au Canada montrent que la numération plaquettaire est élevée en cas de drépanocytose [21]. De ce fait les plaquettes activées sécrètent la thrombospondine (TSP) impliquée dans le pontage globules rouge-endothélium donc une hypercoagulabilité est observée, c'est la cause de la survenue des crises drépanocytaires [16].

Le VGM enregistré chez certains patients est inférieur à la normale, cette microcytose peut être masquée soit par une carence martiale soit due aux âges extrêmes de certains sujets. Le taux d'hémoglobine a été plus faible que celui observé chez les Européens et pourrait être expliqué par des facteurs environnementaux et sociaux économiques [22]. Les troubles hématologiques observées varient selon l'importance de déficit. Ils sont la conséquence du défaut de production d'hémoglobine par défaut de synthèse des chaînes. Ces troubles sont discrets chez les sujets hétérozygotes mais toujours très grave chez l'homozygote. Les hémoglobinopathies identifiées sont classées par fréquence décroissante comme suit: Les drépanocytoses (hétérozygotes et homozygotes) puis les traits ß-thalassémies puis l'hétérozygotie composite S/ß-thalassémie et enfin l'hémoglobinose C et l'hétérozygotie composite S/C et un cas d'a thalassémie. Des études antérieures, ont mis en évidence d'autres types d'anomalies de l'hémoglobine (thalassémies) [ 1, 2, 19], ce qui laisse supposer une prévalence encore plus importante des hémoglobinopathies. Nos résultats ne diffèrent pas des études antérieures notamment celle faite par Alami sur la prévalence des hémoglobines anormales au Maroc, a montré que le gène S a une forte prévalence dans la région de Rabat Salé Kenitra [23]. L'amélioration globale de la prise en charge passe par la mise en place d'un système efficient de protection sociale dans notre pays pour toutes les couches de la société et la mise en place du programme national de prévention et de contrôle de la thalassémie et des autres hémoglobinopathies. En effet le succès d'implantation de ce programme est tributaire d'un leadership qui devrait être fort et participatif, axé sur un soutien des gestionnaires, renforcé par l'allocation appropriée de ressources, soutenu par une communication adaptée aussi bien aux besoins des soignants que de ceux des patients, de leur familles et du grand public, de la formation et l'implication des professionnels de santé pour favoriser leur engagement, de la mise en place d'un système d'information pour la coordination des activités des différents niveaux de soins, du renforcement du rôle du centre de référence et enfin d'un financement approprié pour mettre en place un programme de dépistage des porteurs sains.

## Conclusion

Ce travail porte sur une étude effectuée sur 379 cas suspects d'hémoglobinopathies de la population de la région de Rabat Salé Kenitra. Il nous a permis de démontrer que la prévalence des hémoglobinopathies dans la région de Rabat Salé Kenitra est 72.6 % par rapport à la population étudiée. On a détecté l'existence de différents types d'anomalies d'hémoglobine et leur répartition géographique. Toutes ces anomalies coexistent dans la même population et les formes combinées sont loin d'être rare. L'intérêt de l'étude de ces pathologies est le dépistage des porteurs et la prévention des hémoglobinopathies.

### Etat des connaissances actuelle sur le sujet

- Les hémoglobinopathies sont des matériels génétiques du sang dues à la transmission héréditaire de gènes mutants responsables de la synthèse de l'hémoglobine provenant de deux parents généralement en bonne santé;
- On estime qu'il naît chaque année dans le monde, et en majorité dans les pays à revenu faible ou moyen, plus de 300 000 enfants présentant une forme grave d'hémoglobinopathie;
- Environ 5% de la population mondiale sont des porteurs sains d'un gène drépanocytaire ou thalassémique; ce pourcentage atteint 25% dans certaines régions.

### Contribution de notre étude à la connaissance

- Contribuer à une meilleure prise en charge des familles touchées par ces désordres et contribuer à l'aide des décideurs à proposer des stratégies appropriées de prévention et de prise en charge de ces pathologies;
- Encourager et faciliter la recherche en vue d'améliorer la qualité de vie des malades.

## Conflits d'intérêts

Les auteurs ne déclarent aucun conflit d'intérêt.
